# Mechanisms and predictors of menses resumption once normal weight is reached in anorexia nervosa

**DOI:** 10.1186/s40337-023-00893-x

**Published:** 2023-09-29

**Authors:** Bogdan Galusca, Aurélia Gay, Gwenaëlle Belleton, Martin Eisinger, Catherine Massoubre, François Lang, Dominique Grouselle, Bruno Estour, Natacha Germain

**Affiliations:** 1 Division of Endocrinology, Endocrinology Department, University Hospital of Saint-Etienne, 42055 Saint-Étienne Cedex 2, France; 2 EA 7423, Eating Disorders, Addictions and Extreme Body Weight Research Group, Saint-Étienne, France; 3 Eating Disorder Reference Center of Saint-Etienne, University Hospital of Saint-Etienne, Saint-Étienne, France; 4 Division of Psychiatry, University Hospital of Saint-Etienne, Saint-Étienne, France; 5 UMR 894 INSERM Psychiatry and Neurosciences Center, Paris Descartes University, Paris, France

**Keywords:** Recovered anorexia nervosa, Menses resumption, Body weight set-point, LH pulse, Predictive markers

## Abstract

**Background:**

In cases of Anorexia Nervosa (AN), achieving weight gain recovery beyond the lower limits set by the World Health Organization and normalizing classical nutritional markers appears to be essential for most patients. However, this is not always adequate to restore menstrual cycles. This discrepancy can cause concern for both patients and healthcare providers, and can impact the medical management of these individuals. Thus, the purpose of this study was to assess the ability of anthropometric and hormonal factors to predict the resumption of menstrual cycles in individuals with anorexia nervosa upon reaching a normal body weight.

**Method:**

Patients with AN who had achieved a normal Body Mass Index but had not yet resumed their menstrual cycles (referred to as ANRec) were evaluated on two occasions: first at visit 1 and then again 6 months later, provided their body weight remained stable over this period (visit 2). Among the 46 ANRec patients who reached visit 2, they were categorized into two groups: 20 with persistent amenorrhea (PA-ANRec) and 26 who had regained their menstrual cycles (RM-ANRec). Anthropometric measurements, several hormone levels, Luteinizing Hormone (LH) pulsatility over a 4-h period, and LH response to gonadotropin-releasing hormone injection (LH/GnRH) were then compared between the two groups at visit 1.

**Results:**

Patients in the RM-ANRec group exhibited higher levels of follicular stimulating hormone, estradiol, inhibin B, LH/GnRH, and lower levels of ghrelin compared to those in the PA-ANRec group. Analysis of Receiver Operating Characteristic curves indicated that having ≥ 2 LH pulses over a 4-h period, LH/GnRH levels ≥ 33 IU/l, and inhibin B levels > 63 pg/ml predicted the resumption of menstrual cycles with a high degree of specificity (87%, 100%, and 100%, respectively) and sensitivity (82%, 80%, and 79%, respectively).

**Conclusions:**

These three hormonal tests, of which two are straightforward to perform, demonstrated a high predictive accuracy for the resumption of menstrual cycles. They could offer valuable support for the management of individuals with AN upon achieving normalized weight. Negative results from these tests could assist clinicians and patients in maintaining their efforts to attain individualized metabolic targets.

***Trial registration*:**

IORG0004981.

**Supplementary Information:**

The online version contains supplementary material available at 10.1186/s40337-023-00893-x.

## Abstract (plain language summary)

Once a minimally normal weight has been reached during eating disorder recovery for female patients with anorexia nervosa (AN), the persistence of amenorrhea can be a cause for concern both patient and practitioner. In our study, we have discovered that positive results in biological blood tests, which can be conveniently conducted in an ambulatory setting, offer valuable predictive insights. Specifically, parameters such as LH pulse numbers exceeding 2, LH response to GnRH injection surpassing 33 UI/L, or Inhibin B levels in the blood exceeding 63 pg/mL, can accurately predict the resumption of menstrual cycles in the upcoming months, provided that the patient does not experience weight loss or engage in intense exercise. Conversely, negative results from these tests at this critical juncture in the recovery process can serve as valuable tools to encourage and motivate both the healthcare provider and the patient. By maintaining their efforts and continuing to increase their weight, patients can work towards a more comprehensive restoration of their menstrual cycles.

**Supplementary Information:**

The online version contains supplementary material available at 10.1186/s40337-023-00893-x.

## Background

Anorexia Nervosa (AN) is an eating disorder characterized by self-starvation leading to weight loss, undernutrition, and subsequent adaptive typical hormonal changes [1, 2]. Some of these changes are responsible for a functional gonadal axis blockade [3]. Specifically, the combination of weight loss, blunted leptin [4], high cortisol [5] and high ghrelin plasma levels [6, 7] results in a functional hypothalamic amenorrhea through the inhibition of GnRH pulses [8, 9]. Consequently, amenorrhea was systematically associated with weight loss in the previous DSM IV definition of AN [10, 11]. Nevertheless, one major modification included in the revised DSM 5 definition was the removal of amenorrhea [12], potentially impacting the differential diagnosis of thinness [13].

However, resumption of menstrual cycles during weight gain remains highly relevant for nutritional recovery [10]. While achieving weight gain beyond the lower limits of the World Health Organization's body mass index (BMI) normal range (18.5 kg/m^2^) and normalizing disrupted nutritional markers is generally considered essential, it does not always guarantee the return of menstruation in patients with AN [3]. It has been observed that menses typically resume within six months after reaching 90% of standard body weight for high and age [14]. Meanwhile the delay of menses resumption after weight recovery appears to vary from 6 months [14] to several years [15]. However specific individual set-point of weight and body composition enabling menses recovery can be experienced in clinical practice. Additionally intense physical activity may contribute to amenorrhea persistence despite BMI normalization [16]. Consequently, patients with AN and healthcare professionals alike require well-defined weight gain and physical activity targets, as menses recovery appears to be influenced by factors beyond BMI normalization alone.

Several parameters, including initial and current Follicular Stimulating Hormone (FSH), inhibin B, and anti mullerian hormone (AMH) plasma level [17, 18], baseline cortisol plasma level [19], body weight before the onset of anorexia episode, duration of the illness, and the rate of weight gain [20, 21], entered multivariate models to predict menses recovery in the referred studies. However, these studies have not examined the absolute values of these parameters as thresholds or benchmarks to predict menses restoration and propose them as reliable indicators that could impact clinical decision-making.

Considering the collective insights derived from these observations, the focus of this study was to investigate the potential impact of both anthropometric and hormonal factors in the persistence of amenorrhea among individuals with anorexia nervosa who have achieved normalized weight. Specifically, the study aimed to explore whether these factors play a role in the mechanisms underlying the continued absence of menstruation and to assess their potential to predict the likelihood of subsequent resumption of menstrual cycles.

## Methods

### Ethics

This study was conducted in accordance with the ethical standards of the 1964 Declaration of Helsinki and its subsequent amendments (as revised in 1983). The study received approval from the local Research and Ethics Committee of Saint-Etienne, France (IORG0004981). All subjects provided written informed consent.

### Patients and study design

**Fig. 1:**
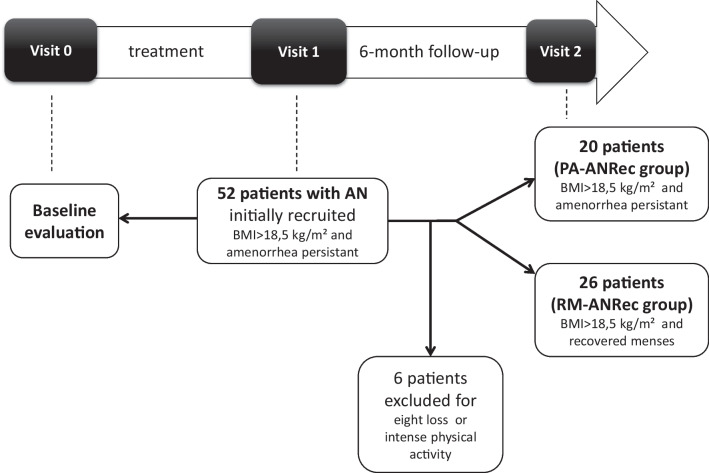
Study design and flow chart of the study

This was a prospective observational study. The study design and flow chart is presented Fig. 1.

Female patients with restrictive-type anorexia nervosa (AN) included in the study analysis met the criteria of the DSM IV and 5 [11] at visit 0. The minimal period between puberty onset and AN onset was two years; hence, no patient with primary amenorrhea was included. None of the patients used oral contraceptives throughout the study period. None of these subjects had other documented chronic or congenital diseases, and none were taking any medication at visits 0, 1 and 2 or between visits 1 and 2. Patients with AN with a documented history of constitutional thinness were not included. Patients were followed in an Eating Disorder Reference Center, under the care of both an endocrinologist/nutritionist and a psychiatrist. From visit 0 to visit 1, the primary somatic goal was to assist patients in gaining weight. From visit 1 to visit 2, primary somatic goal was weight maintenance, with no addition of physical activity.

Initially, 52 female patients with AN were recruited from the Eating Disorder Reference Center of Saint-Etienne when they reached a BMI superior to 18.5 kg/m^2^ during weight gain but still experiencing amenorrhea (ANRec patients) (visit 1). They were then evaluated 6 months later to determine whether amenorrhea persisted despite body weight stabilization (visit 2). Six ANRec patients were excluded due to reported intense physical activity or weight loss during this period, indicating relapse. Consequently, 46 ANRec patients were ultimately included in the study and categorized into two groups at the end of this 6-month follow-up period: 20 patients with persistent amenorrhea despite weight gain preservation, termed Persistent amenorrhea ANRec (PA-ANRec); and 26 patients who recovered their menses during the 6-month follow-up period, termed Recovered Menses ANRec (RM-ANRec). A procedure timeline for data collection is presented in Table 1. Historical maximal BMI (before the disease occurred) was also gathered by self-report.

**Table 1:** Procedure timeline for data collection at visit 0 (before therapeutic intervention), visit 1 (after treatment, weight recovered but persistent amenorrhea) and visit 2 (6 months later)

	Visit 0	Visit 1	Visit 2
Age	x	x	x
BMI	x	x	x
Fat mass	x	x	
Hormonal assessment: plasma FSH, LH, oestradiol, testosterone, SHBG, ACTH, cortisol, GH, prolactin, leptin, free T3, IGF1, SDHA, methoxyamines	x	x	
LH-RH stimulation test		x	
LH pulsatility test		x	
Inhibin B, ghrelin		x	

### Anthropometry and body composition

Body weight was measured with a digital scale (ProDoc, Detecto, PD200M) to the nearest 0.1 kg, and body height was recorded with a standard wall-mounted stadiometer to the nearest 0.1 cm. Fat mass (FM) measurements were assessed by Dual-energy X-ray Absorptiometry (LUNAR, DPX-L).

### Hormonal assessment

Venous blood samples were collected on dry glass tubes containing EDTA, centrifuged, and plasma was aliquoted and kept frozen at − 80 °C before the assay. After an overnight fast, venous blood samples were obtained at 08:00 h for measurement of serum Insulin Like Growth Factor type 1 (IGF-1), Prolactin, TSH, free T3, normetanephrin, metanephrin, Estradiol, FSH, LH, total testosterone, sex hormone binding globulin (SHBG), dehydroepiandrosterone sulfate (DHEAS) and inhibin B. Samples were collected every four hours over a 24-h period (08 h–12 h–16 h–20 h–24 h–04 h) to assess Growth hormone (GH), ACTH, cortisol, and leptin, both acylated and total ghrelin. The assessment techniques of these parameters were previously described [22–25].

FSH and LH responses were also assessed 30 min after intravenous Gonadotropin Releasing Hormone (GnRH) administration (Relefact® LH-RH 100 µg/1 ml, Sanofi Aventis).

LH pulsatile activity was evaluated by blood sampling every ten minutes over four hours in the morning, a day-time known to have the highest frequency of LH pulses.

### Statistical analysis

Hormonal values were presented as mean ± SEM. For GH, cortisol, leptin and ghrelin, the mean value of six circadian assessments was determined for each subject. The time series of LH concentrations over the four hours (LH pulsatile activity test) was analyzed for pulse frequency, pulse amplitude and four hours mean concentration using the validated, objective pulse detection algorithm Autodecon [26]. An LH pulse was defined (and counted) as a significant rise of LH from basal levels every 60 to 90 min, significant enough to be detected within deconvolution analysis (Autodecon).

The two groups, PA-ANRec and RM-ANRec, according to the study design, were compared for all the parameters described in Table 2 (retrospective comparison for visit 0 and 1), BMI at visit 2, and BMI prior to the onset of the disease. For each time point of the study, comparisons between groups were performed using a non-parametric Mann Whitney unpaired t-test. A two-factor repeated measures ANOVA, followed by post hoc test if relevant, was used to compare total and acylated ghrelin during 6-point cycle over 24-h evaluation. A non-parametric Wilcoxon signed rank paired test was used to evaluate changes in all parameters between the time points of the study (visit 1 vs. visit 0).

**Table 2:** Anthropometric and hormonal parameters before and after treatment in the two groups of the study: Persistent amenorrhea group (PA-ANRec) and in Recovered menses group (RM-ANRec)

	Visit 0 (baseline, before therapeutic intervention)			Visit 1 (weight recovered but persistent amenorrhea)				
	PA-ANRec group	RM-ANRec group	*P* value	PA-ANRec group	Time *P* value for PA-ANRec group	RM-ANRec group	Time *P* value for RM-ANRec group	*P* value between groups
Age (years)	20.9 ± 1.0	19.5 ± 1.2	0.44	23.0 ± 0.9	< 0.0001	20.9 ± 1.1	< 0.0001	0.06
BMI (kg/m^2^)	14.9 ± 0.5	15.7 ± 0.3	0.23	18.7 ± 0.2	< 0.0001	19.1 ± 0.2	< 0.0001	0.17
Mean 24-h ACTH (ng/l)	15 ± 1.4	15 ± 1.4	0.96	16 ± 2.4	0.39	11 ± 0.9	0.08	0.05
Mean 24-h Cortisol (nmol/l)	437 ± 39	402 ± 17	0.39	261 ± 17	< 0.0001	232 ± 13	< 0.0001	0.19
Mean 24-h Leptin (µg /l)	2.9 ± 0.2	2.5 ± 0.2	0.05	9.6 ± 0.3	< 0.0001	10.3 ± 0.7	< 0.0001	0.52
Median 24-h GH (mU/l)	21 ± 5	20 ± 0.6	0.56	12.8 ± 8.6	0.40	11.6 ± 4.9	0.31	0.89
Prolactin (mU/l)	288 ± 24	284 ± 18	0.12	230 ± 14	0.005	251 ± 16	0.84	0.35
Free T3 (pmol/l)	2.5 ± 0.1	2.8 ± 0.1	0.11	4.9 ± 0.9	0.04	6.4 ± 0.9	0.006	0.38
IGF1 (µg/l)	148 ± 24	166 ± 17	0.57	254 ± 18	< 0.0001	287 ± 22	< 0.0001	0.28
SDHA (µg /l)	1051 ± 1203	1263 ± 180	0.44	843 ± 162	0.02	1106 ± 101	0.01	0.15
Normetanephrin	1073 ± 132	985 ± 106	0.61	995 ± 120	< 0.0001	810 ± 76	< 0.0001	0.18
Metanephrin	676 ± 65	669 ± 95	0.94	569 ± 74	0.08	545 ± 59	0.35	0.80
FSH (U/l)	4.4 ± 1.2	3.3 ± 0.7	0.44	4.5 ± 0.4 *	0.39	6.5 ± 0.4	< 0.0001	0.001
LH (U/l)	1.9 ± 0.7	0.8 ± 0.2	0.09	3.6 ± 1.2	0.0004	7.2 ± 1.0	< 0.0001	0.04
LH 30’ (U/l)	–	–		17.4 ± 2.5 *	–	54.5 ± 8.7	–	0.0004
Estradiol (ng/l)	16.7 ± 2.7	10.9 ± 1.2	0.17	24.3 ± 4.5 *	0.07	31.4 ± 6.2	0.008	0.39
Total testosterone (ng/dl)	55 ± 9	46 ± 4	0.33	43 ± 4	0.31	52 ± 3	0.14	0.07
SHBG (nmol/l)	92 ± 13	96 ± 10	0.80	59 ± 7	0.02	59 ± 5	0.001	0.97
Inhibin B (pg/ml)	–	–	–	42 ± 7 *	–	69 ± 6	–	< 0.0001

Data are presented as mean ± SEM

* P values less than 0.05 were considered statistically significant

ROC curve analysis was performed to assess sensitivity, specificity, and accuracy (Area under the curve–AUC) of different hormonal markers at visit 1 to predict menses resumption during the 6-month follow-up period.

Statistical analyses were performed with StatView 4.5 software (Abacus Concepts, Inc., Palo Alto, CA).

## Results

### Patients’ overall group characteristics

The mean age of total group at visit 0 was 20.0 ± 0.86 years. The mean maximal BMI prior to disease was 19.9 ± 0.3 kg/m^2^. It decreased at Visit 0 (first admission) to a mean value of 15.1 ± 0.33 kg/m^2^. At visit 1 (weight normalization) mean BMI increased to 18.9 ± 0.15 kg/m^2^. The average time elapsed between visit 0 and visit 1 was 1.6 ± 0.23 years. By the end of the study (visit 2, 6-month follow-up) the mean BMI was 19.2 ± 0.38 kg/m^2^.

### Anthropometric and hormonal inter-group comparison at visit 0 (undernutrition state)

All anthropometric and hormonal parameters are presented in Table 2.

At visit 0, no major differences were noticed between groups while patients were undernourished. Gonadal axis evaluation showed no difference between the groups at visit 0.

BMI was similar between groups at visit 0 (Table 2) but also before the AN diagnosis (19.7 ± 0.4 kg/m^2^ in PA-ANRec vs. 20.3 ± 0.5 kg/m^2^ in RM-ANRec, *p *= 0.6754). The decrease of BMI at this visit (visit 0 BMI–BMI prior to disease) was not different between the groups (− 5.2 ± 0.51 vs. − 4.5 ± 0.54 kgM^2^, *p *= 0.36).

### Anthropometric and hormonal comparison at visit 1 (weight normalisation) and between visit 0 and 1

At visit 1, no differences were noticed between groups for BMI. Although increased when compared to visit 0, BMI at visit 1 was lower than BMI prior to disease. This delta BMI (BMI visit 1–BMI prior to disease) was similar between PA-ANRec and RM-ANRec (− 1.15 ± 0.46 vs. − 0.93 ± 0.37 kg/m^2^, *p *= 0.71).

Lower cortisol and higher leptin, free T3 and IGF1 were found in both groups when compared to visit 0. At visit 1, RM-ANRec patients also presented with higher plasma levels of FSH, LH and inhibin B and higher LH response to GnRH injection compared to PA-ANRec (*p *= 0.0004) (Table 2).

LH pulse analysis performed only at this visit showed significantly lower peak frequency (0.8 ± 0.2 vs. 2.0 ± 0.2 peaks/24 h; *p *= 0.005), area under the curve of LH (639 ± 145 vs. 1067 ± 139; *p *= 0.04), and mean LH (2.4 ± 0.5 vs. 4.1 ± 0.5 U/l; *p *= 0.04) and significantly higher inter-pulse interval (686 ± 94 vs. 313 ± 73; *p *= 0.003) in PA-ANRec patients compared to RM-ANRec patients. Examples of individual LH pulse deconvolution in a PA-ANRec patient and a RM-ANRec patient are presented in Additional file 1: Fig. S1.

Both total and acylated ghrelin plasma levels evaluated at visit 1 (BMI normalization time-point) were significantly higher in PA-ANRec patients compared to RM-ANRec patients (respectively *p *= 0.003 and 0.002) (Additional file 2: Fig. S2).

The time spent between Visit 0 and Visit 1 trended to be higher in PA-ANRec than in RM-ANRec (2.10 ± 0.41 vs. 1.35 ± 0.22 yrs, *p *= 0.06). The mean time elapsed between visit 1 and menses resumption in RM-ANRec group was 3.71 ± 0.51 months.

### Anthropometric comparison at visit 2

Similar BMIs were also found in PA-ANRec and in RM-ANRec by the end of the study (19.0 ± 0.12 vs. 13.3 ± 0.23, *p *= 0.75). No significant differences were found when compared to visit 1 in both groups.

### ***ROC analysis to predict menses recovery (***Fig. 2***)***

**Fig. 2:**
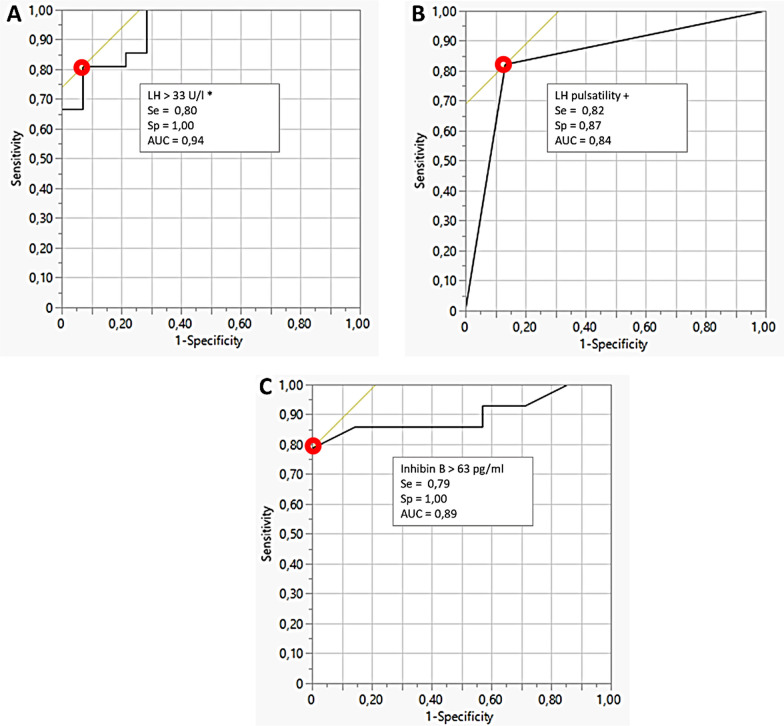
Receiver Operator Curve (ROC) analysis for A LH response to GnRH, B Presence of LH pulses and C inhibin B plasma level; Optimal values of sensitivity (Se), specificity (Sp) and accuracy (AUC) corresponding to the red-circle point on the ROC curve are presented

ROC analysis indicated that LH response to GnRH (≥ 33 UI/l) (Fig. 2A), the number of LH pulses ≥ 2 (Fig. 2B), but also Inhibin B > 63 pg/ml (Fig. 2C) showed equivalent high values of sensitivity (79 to 82%) and specificity (87 to 100%) with AUC around 0.90 to predict menses recovery. Estradiol levels, one-point levels of LH, and FSH predicted menses resumption with lower accuracy.

## Discussion

The persistence of amenorrhea following the attainment of a minimally normal weight during the recovery phase poses a significant challenge for both patients with anorexia nervosa and healthcare professionals. The mechanisms contributing to this blockade or dysfunction of the gonadotropic axis appear to be multifaceted, involving various hormonal factors.

Despite normalization of BMI in all patients of this study, our study clearly demonstrates the existence of two distinct populations of patients with regards to gonadal axis functioning which corresponds to their clinical evolution. Indeed, patients with anorexia nervosa who recovered their weight and further on their menses, displayed higher plasma levels of FSH, LH and inhibin B. Moreover, they presented with higher LH response to GnRH injection. Furthermore, LH pulse analysis showed higher peak frequency, higher area under the curve of LH, higher mean LH, and higher inter-pulse interval, indicating a more “awakened” gonadal axis than in those who did not recover their menses during the follow-up period. Remarkably, these differences occur despite the disappearance of undernutrition signs in both groups. Indeed, nutritional parameters such as free T3 and IGF1 reached similar normalized levels in both studied anorexia nervosa groups. Taken together, these findings suggest that other hormonal parameters involved in gonadal axis functioning could explain the differences observed in both biological and clinical evolution between the two studied groups.

Hormones such as leptin, cortisol and prolactin do not seem to contribute the persistence of amenorrhea after weight normalization. Notably, all patients at visit 1 displayed normalized values for leptin, cortisol, and prolactin. While low leptin levels and functional hypercortisolism are known to underlie hypothalamic amenorrhea in undernourished patients with AN, and the administration of recombinant leptin has been shown to restore menses in women with hypothalamic amenorrhea and leptin deficiency [27, 28] the normalization of these hormones post-weight recovery implies that they might not be directly associated with the ongoing amenorrhea in this context. It is plausible that other hormones or neuropeptides could be involved.

In our study, circadian levels of acylated and total ghrelin were significantly higher in weight normalized patients with anorexia nervosa with persistent amenorrhea than in those with subsequent menses resumption. An increased level of ghrelin in restrictive-type anorexia nervosa with secondary amenorrhea have been widely established [29]. These higher levels could account for a persistent negative metabolic balance explained either by undeclared excess of physical activity or by insufficient food intake. This aligns with previous research suggesting that increased ghrelin levels could act as a metabolic signal hindering the return to a cyclic state in women with eating disorders who exhibit normal weight and body fat but still experience amenorrhea [7]. Moreover, recent evidence highlights ghrelin inhibitory effects on GnRH pulse activity and LH secretion in both animal and human models [6, 30, 31].

Brain imaging data we previously published showed an increased opioid activity in hypothalamic and pituitary areas in undernourished patients with anorexia nervosa when compared to healthy volunteers. In the same study, patients with AN with recovered gonadal activity after weight gain presented with normal opioid activity in these areas, significantly lower than in undernourished patients [32]. Endogenous opioids and their antagonists modulate GnRH pulse and LH secretion [33]. In adult men, treatment with morphine resulted in a decrease of LH pulse frequency while administration of naloxone by itself increased the LH pulse frequency [33]. Interestingly, naltrexone is able to induce ovulation in amenorrheic women [34–36]. However in undernourished patients with anorexia nervosa mean levels of LH and LH pulse activity were not significantly changed by naltrexone [37]. We could account here that LH pulse activity restoration in fully recovered patients with anorexia nervosa may be partly due to a decrease in opioid tone. Further interventional studies could address the question of opioid agonist or ghrelin analogue utility in patients with anorexia nervosa with normalized body weight but still amenorrheic.

Collectively, these findings suggest that specific hormonal and neurobiological abnormalities may persist despite BMI normalization or the normalization of classical nutritional markers. These factors could potentially impede complete metabolic recovery and explain the delay in gonadotropic axis recovery.

Interestingly, we previously demonstrated in this very specific population that pulsatile GnRH therapy was a safe and efficient treatment in cases where patients desired to become pregnant, but still did not allowed menses recovery after pregnancy [38]. This suggests the chronic profile of upper mentioned abnormalities and the importance of their impact on gonadal axis. Undeclared features like intense physical activity or psychological stress may be responsible for this chronic profile of incomplete somatic recovery from AN.

We also evaluated in the current study the potential of certain hormones at the stage when patients with anorexia nervosa achieved a normal BMI but remained amenorrheic to predict future menses resumption. Notably, the differences in gonadal axis between the two groups were evident in our study while all patients were still amenorrheic. This distinguishes our study from many prior ones that found differences in gonadal axis and other hormonal parameters between recovered AN subjects with persistent amenorrhea and those who had already resumed menses [17, 19–21]. These previous findings were expected as they compared two clinically distinct conditions. In contrast, our study reveals these differences before clinical changes’ manifest, indicating their potential as predictive markers. In clinical practice, such hormonal evaluations might inform and educate patients about their gonadal status while supporting clinician recommendations to pursue further weight restoration.

For the first time, our study demonstrates that specific parameters of gonadotropic function are reliable tools for predicting menses recovery in weight-normalized but still amenorrheic patients with anorexia nervosa. The presence of at least two LH pulses within a 4-h interval predicts menses resumption accurately within the next 6 months. As LH pulsatile activity is fundamental for gonadotropic function, this test serves as a reference point. Typically, research studies assess LH pulsatile activity over a 24-h span [39–41]. Our study focused solely on detecting this activity within a shorter 4-h period, as our objective was to identify its presence. During puberty onset, the LH pulse initiates during the night and gradually becomes cyclic throughout the day; however, in adults, LH pulse screening is typically conducted in the morning [42] as performed it in our study.

Comparable accuracy was observed for the 30-min LH response to GnRH test (≥ 33 UI/l) and for inhibin B plasma levels (≥ 63 pg/ml). While LH pulse evaluation proved to be reliable in predicting menses recovery, its complexity limits its practicality in routine clinics. In contrast, inhibin B is simpler to sample and assay, and the GnRH stimulation test is commonly performed in outpatient settings, indicating their potential as practical routine tools.

It is crucial to bear in mind that the assessment of these predictive markers would lose its relevance if a patient recovering from anorexia nervosa, who had previously regained weight, were to start losing it again due to disruptions in their energy balance or engaging in intense physical exercise. Simultaneously, our study revealed a trend toward a longer time lapse between visit 0 and visit 1 in the PA-ANRec group. One might question the relevance of this time frame, considering that weight gain is seldom linear. However, it is plausible that patients with AN, experiencing a more prolonged period of amenorrhea and weight gain, may require extra time for menstrual resumption following weight normalization. In such instances, it could prove advantageous to reevaluate the gonadal axis every six months.

Our findings underscore the importance of tailored care during the recovery process for individuals with anorexia nervosa. However, while utilizing individualized BMI growth curves as a benchmark may be the most effective approach [14], it is often impractical. Individual growth BMI curves may not be readily available, especially for post-adolescent young adults. In some cases, these growth curves may be obscured or biased by prior weight fluctuations occurring before the onset of AN. Additionally, this equilibrium set point can change or be influenced by factors such as physical activity. The aim of this work was not to impose a BMI of 18.5 kg/m^2^ at all costs as a weight gain objective but to propose it, once it has been reached (without menses recovery), as a milestone to trigger an assessment of the gonadotropic axis in all these situations. We selected this value of 18.5 kg/m^2^ as it still represents the lower normal limit for BMI recommended by the World Health Organization (WHO), a value familiar to most patients.

## Conclusions

In conclusion, we propose that the presence of at least two LH pulses over 4 h, an LH response to GnRH ≥ 33 UI/l, or inhibin B plasma levels ≥ 63 pg/ml can accurately predict the resumption of menses in weight-normalized but persistently amenorrheic patients with anorexia nervosa, provided there is no weight loss or intense exercise. These evaluations could assist clinicians in patient counseling. Based on this evaluation, clinicians can inform patients if their individual body weight or metabolic set-point has been achieved or not, thereby guiding them towards continued care: extending weight gain to achieve individual body weight equilibrium and subsequent menses resumption, or maintaining weight while awaiting menses to resume.

### Supplementary Information

**Additional file 1: Fig. S1.** Example of deconvolution of LH pulse for **A** a patient from Persistent Amenorrhea Recovered Anorexia Nervosa (PA-ANRec) group and **B** a patient from Recovered Menses Recovered Anorexia Nervosa (RM-ANRec) group at visit 1. The upper graphs present the absolute LH values throughout the LH pulsatility 4-hour test while the lower graphs are issued from the deconvolution analysis. Patient (**A**) present with no pulses while pulses were detected in patient (**B**).

**Additional file 2: Fig. S2.** Six-point circadian plasma levels of **A** Acylated and **B** Total ghrelin in both groups of the study: Recovered Menses AN after weight recovery (RM-ANRec) (open circles) versus persistent amenorrhea after weight recovery (PA-ANRec) (black triangles).

## Data Availability

Data will be made available upon researchers request for meta-analysis.

## Abbreviations

AMH: Anti mullerian hormone
AN: Anorexia nervosa
ANRec: Recovered anorexia nervosa patient
BGP: Ostéocalcine
BMI: Body mass index
DHEAS: Dehydroepiandrosterone sulfate
DSM: Diagnostic and statistical manual
FSH: Follicle stimulating hormone
GH: Growth hormone
GnRH: Gonadotrophin releasing hormone
IGF1: Insulin like growth factor type 1
LH: Luteinizing hormone
PA-ANRec: Recovered anorexia nervosa patient with persistent amenorrhea
RM-ANrec: Recovered anorexia nervosa patient with recovered menses
ROC: Receiver operating characteristic
sCTX: Serum cross laps
SHBG: Sex hormone binding globulin
WHO: World Health Organization
