# Supplementary material for: Mechanisms and predictors of menses resumption once normal weight is reached in anorexia nervosa
Source: J Eat Disord. 2023 Sep 29;11:172. doi: 10.1186/s40337-023-00893-x (PMC10543836; doi:10.1186/s40337-023-00893-x)
Supplement: Supplementary file 1 — Additional file 1: Fig. S1. Example of deconvolution of LH pulse for A a patient from Persistent Amenorrhea Recovered Anorexia Nervosa (PA-ANRec) group and B a patient from Recovered Menses Recovered Anorexia Nervosa (RM-ANRec) group at visit 1. The upper graphs present the absolute LH values throughout the LH pulsatility 4-hour test while the lower graphs are issued from the deconvolution analysis. Patient (A) present with no pulses while pulses were detected in patient (B). [file 40337_2023_893_MOESM1_ESM.pptx]

## Slide 1
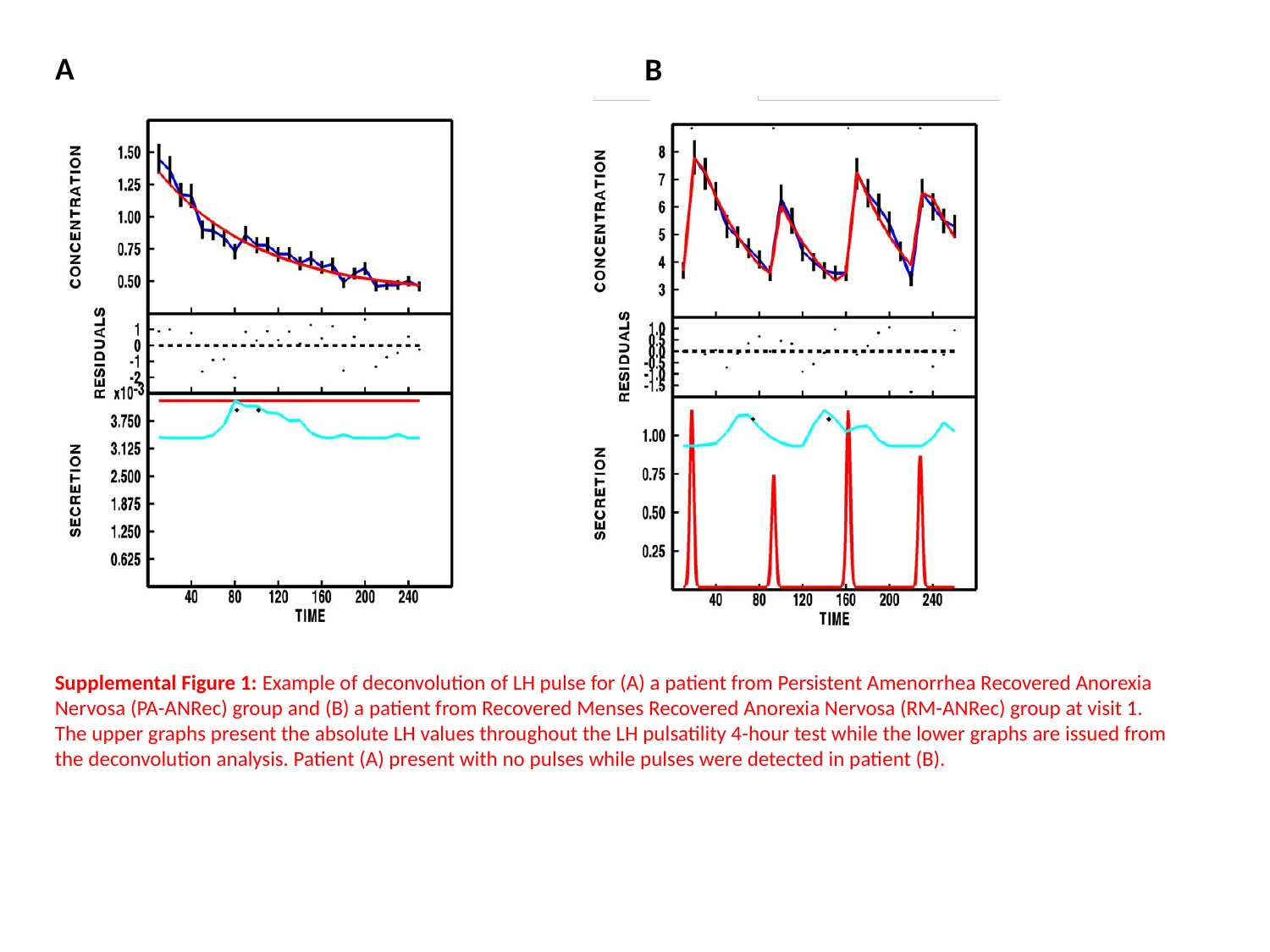

A
B
Supplemental Figure 1: Example of deconvolution of LH pulse for (A) a patient from Persistent Amenorrhea Recovered Anorexia Nervosa (PA-ANRec) group and (B) a patient from Recovered Menses Recovered Anorexia Nervosa (RM-ANRec) group at visit 1. The upper graphs present the absolute LH values throughout the LH pulsatility 4-hour test while the lower graphs are issued from the deconvolution analysis. Patient (A) present with no pulses while pulses were detected in patient (B).
