# Supplementary material for: Mechanisms and predictors of menses resumption once normal weight is reached in anorexia nervosa
Source: J Eat Disord. 2023 Sep 29;11:172. doi: 10.1186/s40337-023-00893-x (PMC10543836; doi:10.1186/s40337-023-00893-x)
Supplement: Supplementary file 2 — Additional file 2: Fig. S2. Six-point circadian plasma levels of A Acylated and B Total ghrelin in both groups of the study: Recovered Menses AN after weight recovery (RM-ANRec) (open circles) versus persistent amenorrhea after weight recovery (PA-ANRec) (black triangles). [file 40337_2023_893_MOESM2_ESM.docx]

**Supplemental Figure 2:** Six-point circadian plasma levels of (A) Acylated and (B) Total ghrelin in both groups of the study: Recovered Menses AN after weight recovery (RM-ANRec) (open circles) vs. persistent amenorrhea after weight recovery (PA-ANRec) (black triangles).
